# Radiological and Functional Assessment of Treatment Outcomes in Patients after Open Reduction with Internal Fixation (ORIF) of Acetabular Fractures

**DOI:** 10.3390/ijerph19031277

**Published:** 2022-01-24

**Authors:** Emilia Dadura, Aleksandra Truszczyńska-Baszak, Dariusz Szydłowski

**Affiliations:** 1 Faculty of Physiotherapy, Józef Piłsudski University of Physical Education in Warsaw, 00-968 Warsaw, Poland; aleksanda.rapala@wp.pl; 2 Department of Orthopaedic Surgery, Professor A. Gruca Independent Public Research Hospital, Centre of Postgraduate Medical Education, 05-400 Otwock, Poland; kor@cmkp.edu.pl

**Keywords:** acetabulum fractures, pelvic, ORIF, functional assessment, HHS, Merle d’Aubigne Scale

## Abstract

(1) Fracture of the pelvis usually happens in young men and results from high-energy trauma. It generates high social and economic costs and results in further health problems. It is therefore important to assess long-term treatment results. (2) The study (NCT04902209) involved 31 patients (mean age 43.6 ± 14.8 years). We conducted fixation assessment on the basis of radiographs and CT scans and functional assessment based on functional scales. (3) We observed more degenerative changes in the less precise reconstruction of the acetabulum (*p* = 0.075). We did not find statistically significant relationships between the area of surgical approach, the gravity of fracture, and the development of degenerative changes. We did not find statistically significant relationships between patients’ functional states and the type of surgical approach or the complexity of the fracture. We found a positive correlation between the time of surgical treatment and patients’ functional state (*p* = 0.04). Patients whose joint surfaces were reconstructed anatomically had significantly higher scores in functional scales (HHS *p* = 0.05, Merle *p* = 0.03). (4) Patients after surgical fixation of the acetabulum have low functional abilities. The quality of reconstruction of the loaded surface as well as the length of time post-surgery seems to be essential for the patients’ functional state.

## 1. Introduction

**Figure 1:**
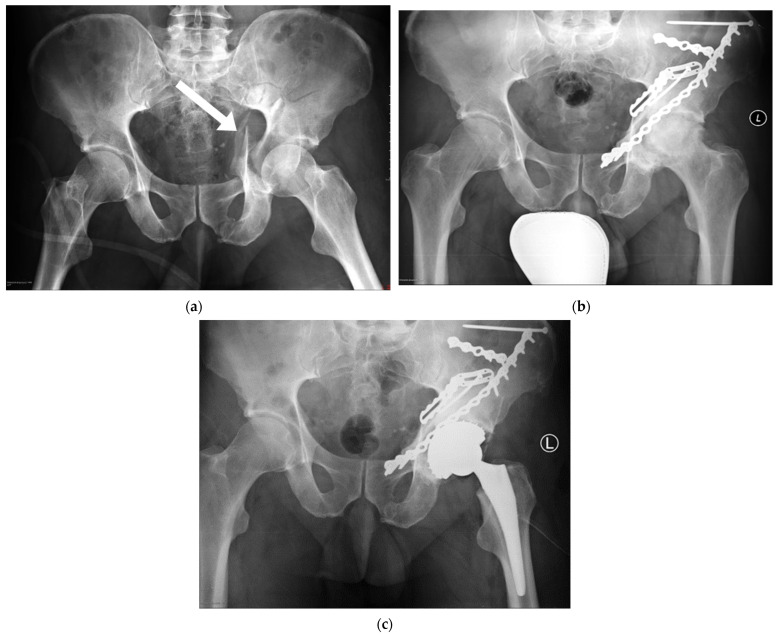
Degenerative changes progression (the arrow—the fracture fissure): (**a**) acetabular fracture (March 2012); (**b**) degenerative changes (April 2015); (**c**) hip arthroplasty (June 2015).

The pelvic ring is usually broken when the forces involved are within the range of 2000–10,000 N [1]. Such forces are involved in high-energy injuries. Road traffic accidents and falls from height are the most common causes of acetabular fractures, as they constitute 76–89% and 7–20% of all such cases, respectively [2,3,4,5,6,7,8,9,10,11]. It is usually young men of working age who are affected [12,13,14,15]. The main region of the hip loaded in the standing position is the roof of the acetabulum (is upper part), and it is in direct contact with the femoral head [16]. Any injury to this area may distort the biomechanics of the joint. This is why surgeons take particular care to reconstruct the greatest possible unbroken area of the loaded surface. Clinical evidence shows that acetabular fractures often result in degenerative changes. Such changes often necessitate early hip arthroplasty (Figure 1). 

Acetabular injuries result in high social and economic costs [12]. Constant monitoring of patients’ health and long-term treatment outcomes analysis is essential. This is why we decided to focus our study on the radiological and functional assessment of treatment outcomes of surgery of acetabular fractures.

## 2. Materials and Methods

### 2.1. The Clinical Group

Prior to the study, we obtained the consent of the Ethics Commission (SKE 01-21/2014), as well as written informed consent of the patients, and registered the trial on ClinicalTrials.gov (NCT04902209). The study was conducted from 2014 to 2017, and it involved 55 patients after open repositioning of acetabular fracture.

The criteria for subject inclusion were the following: age > 18 years, full medical records, the ability to load the operated limb fully, the time after surgery of 3 to 84 months. The follow-up time was determined on the basis of recommendations presented in the literature: three months after surgery, the operated limb should regain its full loading ability [3,4,17,18]; while the incidence of degenerative changes may significantly increase over 84 months post-surgery [19,20], which may significantly affect the functional state of the subjects. All patients were examined during one follow-up visit at a hospital specialist clinic. A revised examination was not possible for organizational reasons, as patients did not always come to control visits.

The criteria for subject exclusion were the following: injuries to the nervous system, laryngological infections, pain and injuries of the spine and lower extremities, chronic diseases (diabetes, Parkinson’s disease, epilepsy, neuromuscular disorders, coronary disease, cancer), and taking psychoactive substances [19,21,22,23].

After applying all the inclusion and exclusion criteria, the study population consisted of 31 patients (24 men and 7 women), whose mean age was 43.6 ± 14.8 years, mean body height was 176.3 ± 8.3 cm, mean body mass was 83.2 ± 16.6 kg, and mean BMI was 26.7 ± 4.9 kg/m^2^. Their injuries resulted from road transport accidents—car accidents (16), motorcycle accidents (4), being hit by a vehicle (2), falls from height (7), and others (2).

All the patients had been surgically treated within 30 days since their accident. Depending on the type of fracture, they had a postero-lateral (24) or ilioinguinal approach. Patients’ functional state was assessed after a mean of 23.6 (± 24.7) months. The fractures were classified according to Judet’s criteria [24]: 17 patients had simple fractures, and 14 patients had complex fractures (Table 1).

All patients were operated on by the same team of experienced orthopedic surgeons alone and were treated according to a unified rehabilitation program by a hospital’s team of physical therapists.

### 2.2. Radiological and Functional Analysis

We conducted patients’ functional state assessment with the use of two questionnaires: the Harris Hip Score (HHS) [25] and the modified Merle d’Aubigne Scale [26]. These are tools of confirmed reliability [Kalariajah, Kirmit, Ugino], and they have been most often used by authors who studied the hip [7,18,27,28]. The maximum HHS score is 100 points, and the maximum Merle d’Aubigne score is 18 points. Higher scores denote patients’ better functional state.

We analyzed patients’ radiographs (AP and transversal) and CT scans with the assistance of an orthopedic surgeon who had had several decades of professional experience. We assessed the quality of fixation and post-traumatic degenerative changes (CareStreamVue PACS), as well as patients’ remaining medical documentation (AMMS System). Following the example of other authors [22,29], we assessed the quality of fixation according to Matta’s criteria [30]. Fracture repositioning with fragment shift of 0–1 mm was considered anatomical, of 2–3 mm—imperfect, and >3 mm was considered poor.

### 2.3. Statistical Analysis

We processed the results with the IBM SPSS Statistics 22.0 program (SPSS Science, Chicago, IL, USA). We applied the Kolmogorow-Smirnow test to verify the normal distribution of the variables. For further analysis, we used non-parametric tests (the distribution was at variance with normal distribution). We calculated correlations between variables using Kendall’s tau-b method. We verified the significance of differences between groups with the Mann–Whitney U-test and chi-squared test. We set statistical significance at *p* ≤ 0.05.

## 3. Results

### 3.1. Radiological Assessment

Of the studied patients, 19 subjects had anatomical fixation. Five of them, despite having had fragments set properly, developed early degenerative changes. Out of 12 patients whose fixation was imperfect, seven developed degenerative changes. The incidence of degenerative changes was higher among patients with poorer acetabulum reconstruction. The statistical values of these differences were close to statistical significance (the chi-squared test 0.075).

We did not find statistically significant relationships between the area of surgical approach and the gravity of the fracture and the development of degenerative changes. This section may be divided into subheadings. It should provide a concise and precise description of the experimental results, their interpretation, as well as the experimental conclusions that can be drawn.

### 3.2. Functional Assessment

Mean values patients scored in both functional scales showed that their functional abilities were poor (Table 2).

The numbers of patients in each HHS/Merle d’Aubigne category were the following (respectively): poor (16/19), fair (7/9), good (5/6), and excellent (3/6). The results from both scales were consistent (Phi = 0.961).

Only three of the studied patients did not suffer from any pain from the operated joint. All the remaining patients reported pain of varying intensity (Table 3).

**Figure 2:**
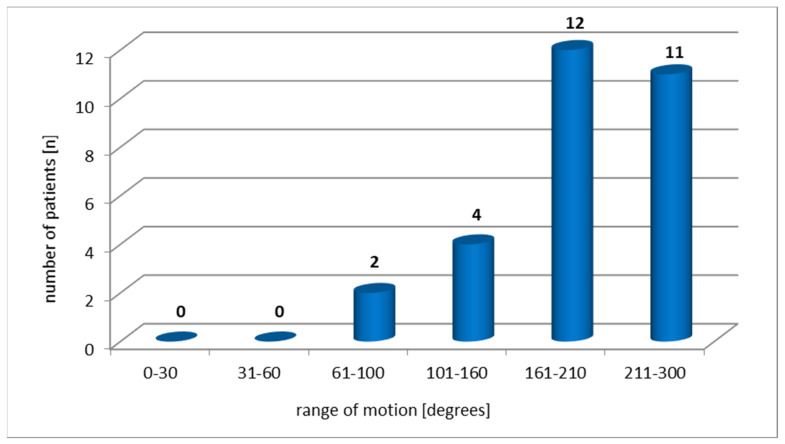
Hip range of motion according to HHS.

The studied patients were found to have a slight limitation to the summary ROM of the hip (Figure 2).

The Kendall’s tau b test did not reveal a statistically significant relationship between the points scored on the functional scales and the age, the gender and the BMI of the studied patients, and the type of surgical approach they had or the complexity of the fracture. We found a positive relationship between the time post-surgery and the scores on the Merle scale (r = 0.27, *p* = 0.04). The longer was the time since the surgery, the better was the physical ability of the patients. The patients whose joints were reconstructed anatomically also had significantly higher scores in both scales (Table 4).

## 4. Discussion

The recent progress in orthopedic surgery has resulted in better long-term surgical treatment outcomes of acetabular fractures. However, such operations are still a big challenge to an orthopedic surgeon [1,16,18]. Matta stated that they resemble “the enigma of the orthopedic surgery” [30]. The analysis of papers written in this area is no easier—scientists analyze different parameters. Authors compare different types of fractures, different types of surgical approaches, and they include or exclude co-existing injuries [31]. All these factors make it more difficult to relate our observations to results by other authors.

The literature stresses that the desirable outcome of acetabular fractures treatment is not only the anatomical reconstruction of the acetabulum and the absence of pain but—first and foremost—regaining the ability of the operated limb. Culemann et al. [14] proved that 8% to 13% of operated patients are likely to have poor functional results.

Our study found a poor functional state in patients after surgical fixation of the acetabulum. Correlating this data with the quality of fixation revealed a positive relationship between the correct reconstruction of the joint surface of the acetabulum and the number of points scored on individual scales. Therefore, the closer the fixation was to the anatomical structure, the better functional state the subjects had (Table 4).

Rommens [1] described the hip as a “non-forgiving joint”—this seems to be very true in the context of an anatomical reconstruction of the acetabulum. He stated that acetabular fractures, especially those that go through its roof, have to be set anatomically. If the optimal reconstruction is not achieved, the patient may suffer from early arthritis, pain of the hip, limited range of movement, or limping [1], all of which definitely affect the level of functional ability. The relationship between the quality of fixation and the patient’s functional state, reported in our material, has also been observed by other authors. A comprehensive systematic literature review presented by Giannoudis et al. [5] confirmed that the key element of positive prognosis of acetabular fracture treatment outcomes was the proper reconstruction of the acetabular roof or the part that transfers loads.

Triantaphillopoulus et al. [27] made a similar statement. They proposed that the joint congruence reconstruction is essential for the functional state. All their patients with anatomical fixation had good or excellent functional states, as measured by HHS [25].

Not all patients with anatomical fixation present an optimal functional state. It is believed that this results from injury-related damage to the joint cartilage (in the loaded area), distortions to the blood supply to the femoral head, or presence of free bodies in the joint, difficult to identify in control radiographs [32,33,34]. There are some opinions in the literature that the experience of the surgical team may be the decisive factor in determining the final functional state of the patient [6,27].

It may seem interesting that some of our patients had poor functional states despite having had anatomical fixation. This relationship shows that a radiograph does not explicitly define a patient’s state. Poor functional state of the patients means that there is a lot of room for therapy.

Our study found a positive relationship between the length of time after surgery and the Merle score (r = 0.27, *p* = 0.04). The longer was the time after surgery, the greater was the physical ability of the patients. This may have been related to the healing of the tissues and with the gradual adaptation to post-operative changes. Our study spanned the period of five years since the injury, so the problem of degenerative changes, which usually develop with time, had not yet intensified. Some authors proved that with the longer follow-up time, the probability of developing degenerative changes increases, even in the case of anatomical fixations [18].

Magu et al. [7] studied a group of 26 patients with anterior wall fracture and found that injuries to the lower limb accompanying the acetabular fracture combined with a BMI > 25 affected patients’ functional results. The follow-up time was 5–22 years. Patients in our study had a mean BMI higher than the norm, yet we did not find the same correlation as Magu et al., possibly because our follow-up time was shorter (five years). Possibly, with time, high BMI would have resulted in the deterioration of the functional state of our patients.

One could assume that complex fractures, as opposed to simple fractures, could lead to poorer functional results. Such tendency was observed by Triantaphillopoulus et al. [27] in their study. Our study, however, did not find such a direct relationship. Still, some authors point out [2,35] that proper reconstruction of joint surfaces is more difficult to achieve in more complex injuries, and this may diminish patients’ functional state. Even though our patients had poor mean functional abilities (Table 2), we found that patients who had poorer quality of fixation scored even fewer points in functional scales (Table 4). One can therefore assume that fracture complexity could indirectly affect the functional state of patients from our study.

The level of functional ability after reconstruction of hip acetabulum should also be considered in a slightly wider context. Schlickewei et al. [36] observed that more than a third of their surgically treated 79 patients had to resign from or significantly limit their sports activity within eight years post-surgery. Giannoudis et al. [22] had similar conclusions. They studied 52 patients and found that after a mean of 3 years of post-operative follow-up period, one-third of their patients did not regain their earlier physical activity.

A study by Nusser et al. [37] on a group of 249 patients with acetabular fracture found that one-third of the subjects lost their ability to return to work within the period of one to two years after completing their physiotherapy. This is potentially a threat to the financial existence of these patients, as well as to their psychological well-being and social integration [37]. The authors agree on the need to provide particularly attentive care and intensive therapeutic programs to those patients who had had acetabular fractures [11,37].

The HHS and Merle scales are useful as they enable authors who study the hip to monitor the treatment outcome. Still, these tools are not perfect [38], so it is important not only to focus on the test scores but also to bear the patients in mind. Moed [33] presented a study in which he discussed a 30-year follow-up of patients after fracture of the posterior wall of the acetabulum. His patients had high Merle scores, yet musculoskeletal function assessment found that they assess their own physical abilities much poorer. Acetabular fractures result in certain functional deficits. This is why it is important to identify the factors that limit patients’ physical abilities and to control their impact on treatment outcomes [33].

### 4.1. Limitations of the Study

The size of the study population was relatively small—this was because there are only a few specialized centers that operate such complicated injuries as pelvic fractures. Also, restrictive inclusion and exclusion criteria played a role here—we needed to minimize the effect of any unrelated parameters on the observed variables. Future studies shall involve a greater number of patients. Still, the observations we made in this study may play a role in determining the focus of future studies on patients with surgical fixation of acetabular fractures.

The variety of fracture types posed a methodological difficulty. To minimize the effect of this variable, we used the complexity of fracture (simple/complex) as a superior criterion for classification. Limiting the scope of the study to one type of fracture would only make the group more homogeneous, yet it would not allow for a broader study of the phenomenon.

### 4.2. The Value of the Study

The study was based on scales of proven reliability [39,40,41], most often used by authors who study the hip. The analysis of radiographs and scans was conducted with the assistance of a very experienced professor of orthopedic surgery, who had had several decades of professional experience. This ensured the reliability and accuracy of classification.

All the patients had been operated on by the same team of highly qualified surgeons working in the same center. This ensured the homogeneity of the group in terms of surgical techniques, tools, and post-operative treatment. To minimize the measurement error related to the person of the researcher, all the study protocols were conducted by the same person.

The analyses spanned a significant period of time. This allowed for observing long-term changes which develop in patients after ORIF. The authors hope that this may draw practitioners’ attention to elements that require particular care in the process of treatment of acetabular fractures of the pelvis. The static and dynamic (gait) analyzes are also needed in these patients, as is the case with hip arthroplasty patients [42].

Future analyses should involve studies assessing the effectiveness of rehabilitation methods used in this group of patients—as Maurer [43] stated, only a proper fixation of the hip accompanied by early and adequate physiotherapy allows for positive treatment outcomes. This seems particularly important from the perspective of the patient.

## 5. Conclusions

1. Patients after surgical fixation of acetabular fractures often have a low level of functional ability.

2. The quality of reconstruction of the loaded surface, as well as time that elapsed since the surgery, seem to be essential for the functional state of patients after ORIF.

## Figures and Tables

**Table 1:** Number of types of fractures according to Judet’s criteria.

Group	Type of Fracture	Number (No.)	∑
Simple	posterior wall	9	17
	posterior column	3	
	anterior wall	1	
	anterior column	2	
	transversal	2	
Complex	posterior column and posterior wall	3	14
	both columns	10	
	T-shaped	1	

**Table 2:** Patients’ functional state—norms.

Scale	Study Population		Functional State—Norms [26,27]			
	$\bar{X}$	SD	Poor	Fair	Good	Excellent
HHS [points]	68.9	16.3	<70	70–79	80–89	90–100
Merle [points]	12.0	2.8	<13	13–14	15–17	18

**Table 3:** Pain intensity according to the HHS.

Pain—HHS	*n*
None, or ignores it	3
Slight, occasional, no compromise in activity	11
Mild pain, no effect on average activities, rarely moderate pain with unusual activity, may take aspirin	11
Moderate pain, tolerable but makes concessions to pain. Some limitations of ordinary activity or work. May require occasional pain medication stronger than aspirin	3
Marked pain, serious limitation of activities	1
Totally disabled, crippled, pain in bed, bedridden	0

**Table 4:** Functional state and the reduction of the fracture.

	Reduction of Fracture				*p* ≤ 0.05 *
	Anatomical		Imperfect		
Scale	$\bar{X}$	SD	$\bar{X}$	SD	*p*-Value
HHS (points)	73.74	15.26	61.33	15.53	0.05 *
MERLE (points)	12.95	2.50	10.58	2.81	0.03 *

* The statistical significance was set at *p* ≤ 0.05.

## Data Availability

The data presented in this study are available on request from the corresponding author. The data are not publicly available due to institutional restrictions.
